# Genome-Wide Expression Profiling of the *Arabidopsis* Female Gametophyte Identifies Families of Small, Secreted Proteins

**DOI:** 10.1371/journal.pgen.0030171

**Published:** 2007-10-12

**Authors:** Matthew W Jones-Rhoades, Justin O Borevitz, Daphne Preuss

**Affiliations:** 1 Department of Molecular Genetics and Cell Biology, The University of Chicago, Chicago, Illinois, United States of America; 2 Department of Ecology and Evolution, The University of Chicago, Chicago, Illinois, United States of America; University of Toronto, Canada

## Abstract

The female gametophyte of flowering plants, the embryo sac, develops within the diploid (sporophytic) tissue of the ovule. While embryo sac–expressed genes are known to be required at multiple stages of the fertilization process, the set of embryo sac–expressed genes has remained poorly defined. In particular, the set of genes responsible for mediating intracellular communication between the embryo sac and the male gametophyte, the pollen grain, is unknown. We used high-throughput cDNA sequencing and whole-genome tiling arrays to compare gene expression in wild-type ovules to that in *dif1* ovules, which entirely lack embryo sacs, and *myb98* ovules, which are impaired in pollen tube attraction. We identified nearly 400 genes that are downregulated in *dif1* ovules. Seventy-eight percent of these embryo sac–dependent genes were predicted to encode for secreted proteins, and 60% belonged to multigenic families. Our results define a large number of candidate extracellular signaling molecules that may act during embryo sac development or fertilization; less than half of these are represented on the widely used ATH1 expression array. In particular, we found that 37 out of 40 genes encoding Domain of Unknown Function 784 (DUF784) domains require the synergid-specific transcription factor *MYB98* for expression. Several DUF784 genes were transcribed in synergid cells of the embryo sac, implicating the DUF784 gene family in mediating late stages of embryo sac development or interactions with pollen tubes. The coexpression of highly similar proteins suggests a high degree of functional redundancy among embryo sac genes.

## Author Summary

During the sexual reproduction of flowering plants, a pollen tube delivers sperm cells to a specialized group of cells known as the embryo sac, which contains the egg cell. It is known that embryo sacs are active participants in guiding the growth of pollen tubes, in facilitating fertilization, and in initiating seed development. However, the genes responsible for the complex biology of embryo sacs are poorly understood. The authors use two recently developed technologies, whole-genome tiling microarrays and high-throughput cDNA sequencing, to identify hundreds of genes expressed in embryo sacs of Arabidopsis thaliana. Most embryo sac–dependent genes have no known function, and include entire families of related genes that are only expressed in embryo sacs. Furthermore, most embryo sac–dependent genes encode small proteins that are potentially secreted from their cells of origin, suggesting that they may act as intracellular signals or to modify the extracellular matrix during fertilization or embryo sac development. These results illustrate the extent to which our understanding of plant sexual reproduction is limited and identifies hundreds of candidate genes for future studies investigating the molecular biology of the embryo sac.

## Introduction

The life cycle of plants alternates between haploid gametophyte and diploid sporophyte generations. A central step in plant sexual reproduction is the transfer of sperm cells from the male gametophyte, the pollen grain, to the female gametophyte, the embryo sac, resulting in fertilization and the formation of a new sporophytic embryo.

In flowering plants, each embryo sac develops within the sporophytic tissues of the ovule, which is itself located within the ovary of the flower. Embryo sac development is preceded by meiosis, and consists of precise series of mitotic divisions, nuclear migrations, cellularizations, and cell deaths (reviewed in [1,2]). In *Arabidopsis*, the mature embryo sac consists of four cells: the egg cell, two synergid cells, and a large central cell. During fertilization, a pollen tube penetrates the sporophytic tissues of the ovule and terminates growth at one of the synergid cells of the embryo sac. Following the rupture of both the targeted synergid and the pollen tube cell, the two sperm cells fuse with the egg cell and the central cell to form the embryo and the endosperm.

Genes expressed within the embryo sac are responsible for many aspects of embryo sac biology. Embryo sac–expressed genes control the developmental program of the embryo sac, as evidenced by the large number of female gametophytic mutants that result in the arrest of embryo sac development at various stages [3–9]. Embryo sac–expressed genes are also central to the fertilization process. Ovules that do not contain functional embryo sacs do not attract pollen tubes [9,10] , an observation which led to the suggestion that the embryo sac produces signals that guide pollen tube growth. Both *Arabidopsis* genetics [11] and in vitro studies with *Torenia* ovules [12,13] have identified the synergid cells as a source of embryo sac–derived pollen tube attractants. In addition, embryo sac–expressed genes are required for the reception of pollen tubes by the synergids [6,14,15] and for the coupling the initiation of seed development to fertilization [16–19].

There has been success in the identification of numerous female gametophytic mutants. However, the total set of genes expressed in the embryo sac is poorly defined due to the fact that the embryo sac is embedded within the sporophytic tissues of the ovule, making it difficult to directly isolate embryo sac tissue for gene expression analysis. Here, we used genetic subtraction to identify embryo sac–expressed genes by comparing gene expression in wild type ovules to that in *determinate infertile1* (*dif1)* and *myb98* mutant ovules. *DIF1* encodes a cohesin required for meiosis; sporophytic tissues of the ovule are unaffected in *dif1* ovules, but gametogenesis is prevented by the failure of meiosis to produce functional megaspores [20,21]. *dif1* ovules therefore represent a clean, genetic ablation of the entire embryo sac. *MYB98* encodes a transcription factor expressed specifically within the synergid cells of the embryo sac [11]. The early stages of embryo sac development in *myb98* ovules resemble wild type, and the only observable morphological differences of mature *myb98* embryo sacs compared to wild type are abnormalities within the subcellular structures of the synergid cells [11]. In addition, *myb98* ovules have an incompletely penetrant pollen tube guidance defect [11]. *myb98* mutations therefore impact the last stages of embryo sac development and specifically impact interaction with pollen tubes.

We used high-throughput (454) cDNA sequencing and whole-genome tiling arrays to compare gene expression in wild-type ovules to that in *dif1* and *myb98* ovules. Importantly, these two techniques allow for genome-wide measurement of gene expression that is unbiased toward annotated genes. We identified 382 genes that were downregulated in *dif1* ovules and 77 genes that were downregulated in *myb98* ovules. The majority of genes downregulated in each mutant belonged to families of small, potentially secreted proteins. Because most embryo sac–dependent genes were unannotated or recently annotated, only 31% were by reported by recent studies of embryo sac gene expression using the annotation-based ATH1 microarray [22,23]. Our results identify a surprisingly large number of embryo sac–expressed, secreted proteins as candidate extracellular signaling molecules during embryo sac development and fertilization, and in particular implicate the poorly understood DUF784 gene family as potential mediators of the last stages of embryo sac development or signaling interactions between the embryo sac and the pollen tube.

## Results/Discussion

### High-Throughput Sequencing of Ovule cDNAs

To obtain an unbiased, genome-wide survey of ovule gene expression, we sequenced stage 14 ovule cDNAs from male sterile *ms-1* plants (Landsberg ecotype) using the high-throughput 454 sequencing method [24]. We obtained 249,440 cDNA reads, comprising a total of 26.5 million bp, with the reads having a median length of 106 bp (Table 1). 225,499 reads (90%) could be confidently aligned to the *Arabidopsis* genome [25] using blat or blastn (Dataset S1), with the majority of unalignable reads consisting primarily of simple sequence repeats and/or PCR primer sequence. 28,732 reads matched equally well to more than one genomic location and thus represent transcripts from recent duplications. Eighty-five percent of alignable reads mapped to annotated exons, covering 12.5% of all annotated exonic sequence. In total, 15,312 annotated genes were matched by at least one cDNA read. The number of reads per gene ranged from 0 to 3,099 (to AtMg00020, a ribosomal protein encoded by the mitochondrial genome), with most genes having 0–5 reads, 15% of genes having ten or more reads, and 5% of genes having 25 or more reads (Figure S1). The aligned cDNA reads were 97.5% identical to the published genome sequence. The 2.5% of bases not matching genomic sequence were likely the result of cDNA sequencing errors as well as ecotype-specific polymorphisms due to the alignment of Landsberg cDNAs to the Columbia genome.

**Table 1:**
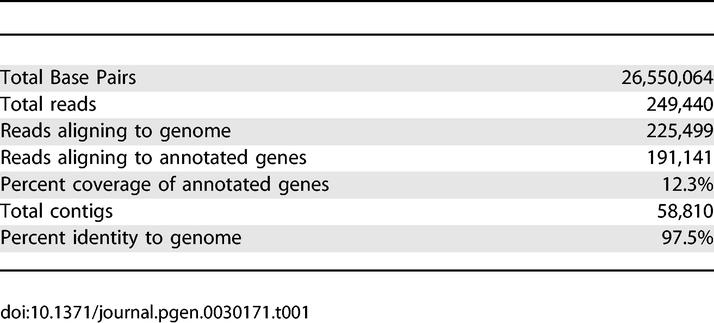
Summary Statistics on Ovule cDNA Reads

### Tiling Array Analysis of Embryo Sac–Dependent Gene Expression

We also searched for embryo sac–dependent transcripts across the entire *Arabidopsis* genome by using whole-genome tiling microarrays to compare gene expression in wild-type (Columbia ecotype) ovules to that in *dif1* and *myb98* ovules. We dissected ovules from mature (stage 14) flowers, collecting sufficient material to yield at least 2 μg of total RNA for each of four biological replicates for each genotype (wild type, *dif1*, and *myb98*), resulting in a total of 12 samples. After reverse transcription, second-strand cDNA synthesis, and double-stranded random labeling, samples were hybridized to Genechip *Arabidopsis* Tiling 1.0F arrays (Affymetrix), which contain over 3,000,000 25mer perfect match probes spread across the *Arabidopsis* genome with a median gap of 10 bp between probes. The log_2_ transformed hybridization signals to the perfect match probes were quantile normalized across the twelve arrays; pairwise correlations ranged from 0.952 to 0.966 (Table S1).

### Identification of Unannotated Embryo Sac–Dependent Genes

Mutations in *DIF1* result in the ovules that entirely lack embryo sacs [20]. To identify embryo sac–dependent transcripts de novo, without bias towards existing gene models, we used a simple algorithm to identify genomic regions differentially expressed between *dif1* and wild-type ovules. In brief, a Welch's *t*-test was performed for each probe comparing the log_2_ scale expression values of the four wild-type replicates to the four mutant replicates. An arbitrary threshold was applied to define probes that correspond to differentially expressed messages (*p* ≤ 0.05, log_2_ fold change ≥ 1). Neighboring probe matches within 80 bp of each other that met this threshold were joined to define differentially expressed intervals, with the requirement that at least three differentially expressed probe matches were required to define an interval.

**Figure 1:**
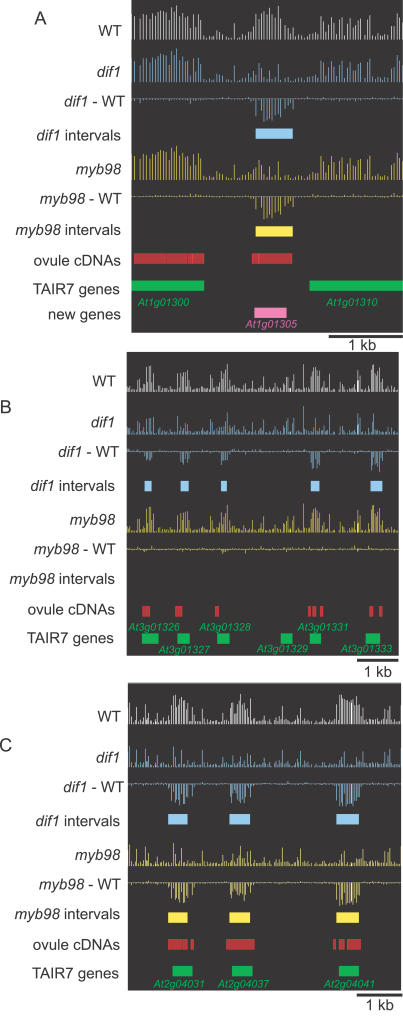
Genomic Regions Differentially Expressed between Wild-Type and *dif1* or *myb98* Ovules The log_2_ transformed probe level expression values are plotted for wild-type (WT, white), *dif1* (blue), and *myb98* (yellow) ovules, as are the probe level differences in expression between wild type and mutant (*dif1* − WT, *myb98* − WT). Genomic intervals identified as differentially expressed in *dif1* (blue) or *myb98* (yellow) are plotted as colored boxes, as are the genomic alignments of ovule DNA fragments (red), previously annotated genes (green), and newly identified ORFs (purple). (A) A genomic interval between the previously annotated genes *At1g01300* and *At1g01310* that is downregulated in both *dif1* and *myb98* ovules and corresponds to a contig of ovule cDNAs is annotated as gene *At1g01305*. Flanking genic and intergenic probes are expressed at similar levels between all three genotypes. (B) A cluster of six DUF1278 genes, five of which are downregulated in *dif1* ovules but not in *myb98* ovules. (C) A cluster of three DUF784 genes that are downregulated in both *dif1* and *myb98* ovules.

Using this algorithm, we identified 1,099 genomic intervals that were downregulated in *dif1* ovules compared to wild type. Of these intervals, 969 mapped to an annotated gene. The remaining 130 seemingly intergenic intervals were compared to the genomic alignments of the ovule cDNAs. After joining adjacent intervals (those separated by less than 200 bp), 27 *dif1* downregulated intervals that overlapped with ovule cDNA alignments were considered as putative unannotated genes. Using cDNA and EST sequences as guides, open reading frames (ORFs) were found for 22 of these putative genes (Tables 2 and S3). Sixteen of the newly identified ORFs were matched by at least three ovule cDNA reads with unique matches to the genome (Table 2). As an example of the array and cDNA data supporting one of the newly annotated genes, a region between the annotated genes *At1g01300* and *At1g01310* with substantially more expression in wild-type ovules than in *dif1* ovules was detected as a differentially expressed interval, whereas surrounding genic and intergenic probes had highly similar expression values between all three genotypes (Figure 1A). In this case, the same interval was also differentially expressed between wild-type and *myb98* ovules, and overlapped with the genomic alignments of 11 ovule cDNAs (Table 1; Figure 1A). The differentially expressed interval also overlapped well with a putative ORF and was assigned the name *At1g01305* (Figure 1A).

**Table 2:**
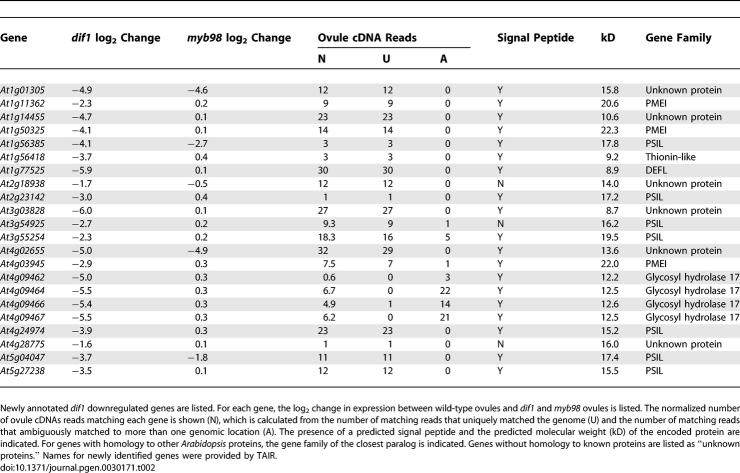
Newly Annotated Embryo Sac–Dependent Genes

### Genome-Wide Analysis of Embryo Sac–Dependent Gene Expression

We used the tiling array data to quantify changes in transcript levels between wild-type and *dif1* ovules for the entire set of *Arabidopsis* genes, including both the 22 genes we identified as well as previously annotated genes (The *Arabidopsis* Information Resource [TAIR] release 7 annotations, containing 27,029 protein coding genes, 3,889 pseudogenes, and 1,123 noncoding RNA genes [http://www.arabidopsis.org]). For each gene, a *t*-test comparing wild-type and *dif1* signal intensities was performed across all probes matching that gene (Table S4). We defined genes as having significantly different expression in *dif1* compared to wild type by setting a *p-*value threshold of 0.001 and a log_2_ fold change threshold of 1 (which corresponds to a 2-fold change). At these cutoffs, we found 382 protein-coding genes that were expressed at lower levels, and 35 genes that were expressed at higher levels in *dif1* ovules (Tables 3, S5, and S6).

**Table 3:**
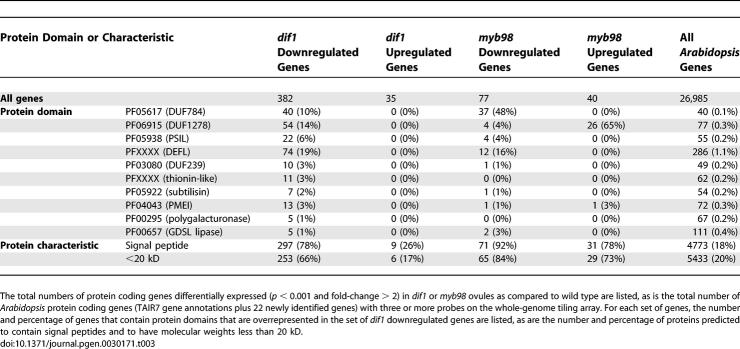
Characteristics of Genes Differentially Expressed in *dif1* or *myb98* Ovules

To empirically assess the extent to which sampling error contributed to the observed differential expression, we estimated the false discovery rate (FDR) by shuffling the eight arrays (four wild type and four *dif1*) into two permuted groups of four and reanalyzed the data to identify the number of genes with seemingly differential expression between these arbitrarily grouped sets or arrays. To control for differences in gene expression relating to the *dif1* phenotype, we considered only the 18 balanced permutations in which the two groups of arrays being compared each contained two wild-type arrays and two *dif1* arrays. On average, 3.6 genes had the *p-*values less then 0.001 and log_2_ changes in expression greater than 1 for the balanced permutations of the wild-type and *dif1* datasets. Therefore, we estimate the FDR to be approximately 1% for the 417 genes differentially regulated between *dif1* and wild type at this threshold. Estimates of the FDR at more relaxed thresholds suggest that several hundred additional genes are differentially expressed in *dif1* ovules with changes in expression less than 2-fold (Table S2).

Because the *dif1* mutation only affects cells that undergo meiosis, the simplest interpretation of these data is that the 382 *dif1* downregulated genes are expressed preferentially within the embryo sac as compared the sporophytic ovule. It is also possible that some of these genes require the presence of the embryo sac for expression within the sporophytic ovule.

### Embryo Sac–Dependent Genes Are Enriched for Families of Small, Potentially Secreted Proteins of Unknown Function

To characterize the set of *DIF1*-dependent genes, we analyzed the abundance of protein domains in the sets of differentially expressed genes as compared to the total set of protein coding genes. Ten gene families were significantly overrepresented in the set of *dif1* downregulated genes (Table 3); 241 of the 382 *dif1-*downregulated genes belonged to one of these ten gene families. Several of these families, such as Domain of Unknown Function 784 (DUF784), DUF1278, and DUF239, lack homology to any protein with a known function. Two families, the Defensin-Like (DEFL) genes and the thionin-like genes, have homology to small, secreted antipathogenic peptides, whereas the *Papaver* Self-Incompatibility-Like (PSIL) genes have homology the pistil-secreted S1 protein of *Papaver*. The functions of these six families within the context of the *Arabidopsis* ovule are unknown. The remaining overrepresented families encode proteins with presumed functions as catalytic enzymes (peptidases, lipases, and polygalacturonases) or as enzyme inhibitors (pectinmethylesterase inhibitors [PMEIs]). Many members of these gene families are encoded by tandemly arrayed, recently duplicated genes (Figures 1B, 1C, S2, and S3).

In addition to the overrepresentation of certain protein domains, the set of *dif1* downregulated genes was highly enriched for genes encoding small proteins that contain putative signal peptides (Table 3). Seventy-eight percent of *dif1* downregulated genes were predicted to encode for a signal peptide, as compared to 18% among all protein-coding genes, and 66% of *dif1* downregulated genes were predicted to encode proteins that weigh less than 20 kilodaltons, as compared to 20% among the total set of annotated proteins (Table 3). This bias towards small proteins with signal peptides was related to the bias towards certain protein families; 91% of the 215 DUF784, DUF1278, PSIL, DEFL, PMEI, and thionin-like genes downregulated in *dif1* encode proteins that contain putative signal peptides and that weigh less than 20 kD. The presence of a signal peptide can target a protein for one of several fates, such as localization to a membrane-bound organelle, localization to the cell membrane, or secretion from the cell. However, the abundance of putative signal peptides amongst *DIF1*-dependent proteins, as well as the fact that numerous *DIF1*-dependent genes have homology to proteins that are known to be secreted in other organisms or tissues (e.g., DEFL, thionin-like, and PSIL genes) [26–28], suggests that many embryo sac–dependent proteins have the potential to act outside of their cells of origin.

The 140 *dif1* downregulated genes that did not belong to the ten gene families listed in Table 2 represented a broad range of functionalities (Table S5). Several *DIF1*-dependent genes have known roles in embryo sac biology, including the synergid-expressed transcription factor *MYB98* as well as two genes, *FERTILIZATION INDEPENDENT SEED2* and *MEDEA,* that regulate the development of the central cell and endosperm [16,17].

**Figure 2:**
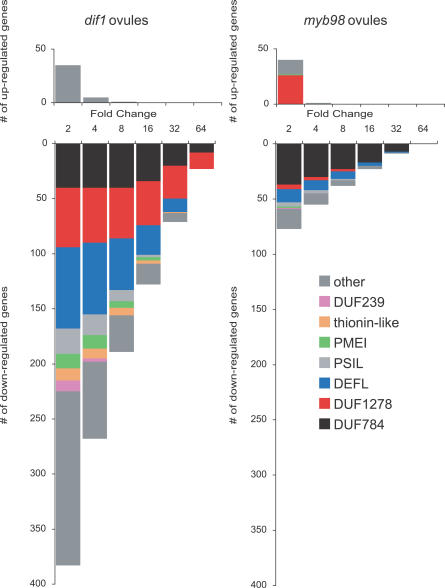
Families of Small, Secreted Proteins Are Highly Downregulated in *dif1* and *myb98* Ovules The number of genes differentially expressed between wild-type ovules and mutant ovules (*p* > 0.001) with changes in expression greater than the indicated cutoffs (2-fold, 4-fold, 8-fold, 16-fold, 32-fold, and 64-fold) are plotted. The numbers of genes belonging to gene families represented by at least ten members among the set of *dif1* downregulated genes are indicated by color coding. (A) Genes differentially expressed between wild-type and *dif1* ovules. (B) Genes differentially expressed between wild-type and *myb98* ovules.

While 382 genes were downregulated at least 2-fold in *dif1* ovules, some genes were more highly downregulated. Most of the genes with large differences in expression levels between *dif1* and wild-type ovules belonged to multigenic families encoding small, potentially secreted proteins (Figure 2). For example, 83% of the 189 genes downregulated at least 8-fold in *dif1* ovules belonged to the DUF784, DUF1278, DEFL, PSIL, PMEI, or thionin-like gene families (Figure 2). All 23 genes that were downregulated at least 64-fold in *dif1* ovules belonged to the DUF784 or DUF1278 families (Figure 2).

**Figure 3:**
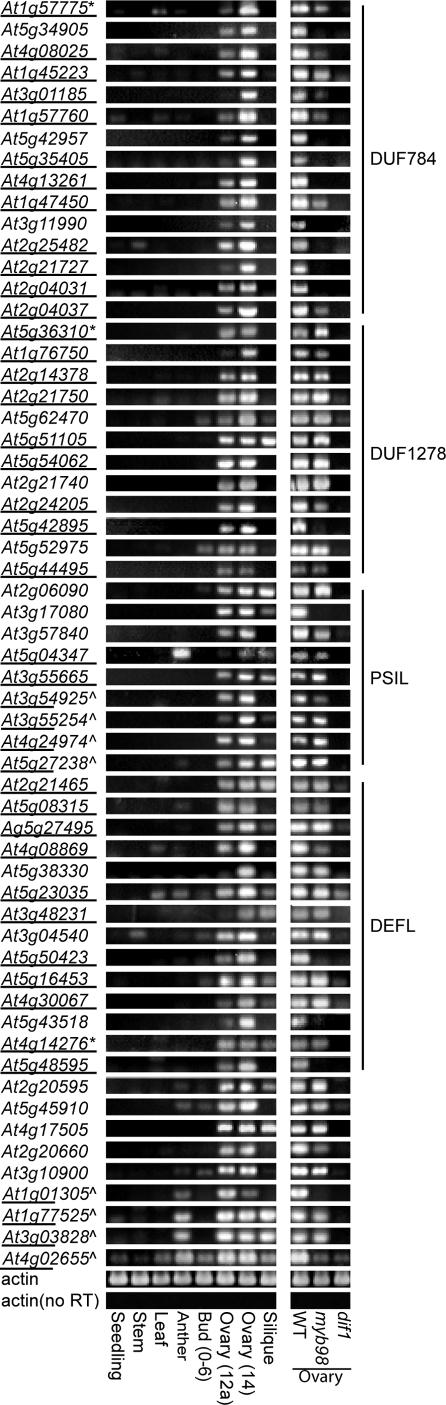
RT-PCR Analysis of Genes Downregulated in *dif1* Ovules Total RNA various *ms-1* tissues, as well as from wild-type (Col) and mutant (*myb98* and *dif1*) ovaries, was used as template for oligo-dT primed reverse transcription, and 1/100th of the first-strand cDNA was used as template per PCR. Underlined genes are not represented on the ATH1 array. Genes marked with a caret are newly annotated by this work. For genes marked with an asterisk, the PCR primers were perfectly complementary to more than one gene with highly similar sequences.

We analyzed the expression patterns of 59 *dif1* downregulated genes by reverse transcriptase PCR (RT-PCR), with a focus on members of the DUF784, DUF1278, PSIL, and DEFL gene families (Figure 3). In all 59 cases, including eight previously unannotated genes, expression was lower in *dif1* ovaries than in wild type (Figure 3).

In addition to 382 protein-coding genes, 26 annotated pseudogenes were also significantly downregulated in *dif1* ovules (Table S9). Most of these pseudogenes had a high degree of homology to adjacent, tandemly arrayed protein coding genes also downregulated in *dif1* ovules (e.g., DUF784 pseudogenes). While some of the observed expression of these pseudogenes may have been due to cross-hybridization to transcripts from homologous protein-coding genes, the fact that 13 were uniquely matched by ovule cDNA reads indicates that many are in fact transcribed (Table S9). It seems that some recently duplicated, embryo sac–dependent genes have retained regulated, functional promoters despite having acquired frame shift or nonsense mutations within their ORFs. It is unclear as to what, if any, functional roles these expressed pseudogenes might play.

### Many Embryo Sac–Dependent Genes Are Highly Similar to Each Other

Many embryo sac–dependent genes have similarity to each other at the nucleotide sequence level, suggesting a common origin and function. The ORFs of 109 *dif1* downregulated genes were >90% identical to another *dif1* downregulated gene. In most cases, highly similar genes were present in tandem arrays of apparently recently duplicated genes. Seventy-five of the *DIF1*-dependent genes could be grouped into 17 clusters of highly similar genes that shared at least 50% of their tiling array probes with another gene. Twenty-six of these partially ambiguous genes could still be identified as significantly downregulated in *dif1* ovules based solely on the expression values of probes with unique matches in the *Arabidopsis* genome. Another 14 were uniquely matched by ovule cDNA fragments, providing evidence that they are expressed in the ovule. Nonetheless, for approximately 50 genes, it is difficult to be certain that the differential expression observed on the tiling array truly reflected the expression of each individual gene or if only a subset of genes were differentially expressed.

The most extreme example of closely related embryo sac–dependent genes is that of 30 DUF1278 genes (as well as three DUF1278 pseudogenes) that are >95% identical to each other. Most probes on the tiling array that correspond to this cluster perfectly match multiple genes; moreover, it was not possible to design RT-PCR primers specific to any particular gene from this cluster. Fifty-six of the 60 cDNA reads matching genes from this cluster matched more than one gene equally well. It is therefore difficult to be certain that the expression observed for genes of this cluster corresponds to all 30 genes or to a subset of the 30 genes. However, the high number of cDNA reads mapping to this genes in this region, together with the high degree (40- to 70-fold) of *DIF1* dependence detected for this region, make it clear that as a unit, this region is expressed in an embryo sac–dependent manner. Moreover, the RT-PCR primers to this region (i.e., to gene *At5g36350*) failed to detect any expression from this cluster in *dif1* ovules despite being perfectly complementary to most of the 30 genes (Figure 3), further demonstrating that no gene from this cluster is highly expressed in ovules that lack embryo sacs.

### Few Genes Are Highly Upregulated in *dif1* Ovules

In contrast to the set of *dif1* downregulated genes, the 35 genes upregulated in *dif1* ovules were not significantly enriched for any protein domains, nor for genes predicted to encode proteins with signal peptides or weighing less than 20 kD (Tables 3 and S6). Furthermore, the magnitude of upregulation was modest compared to changes in expression levels observed for *dif1* downregulated genes. Only one gene was upregulated more than 8-fold in *dif1* ovules (Figure 2).

### Embryo Sac–Dependent Genes Are Poorly Represented on the ATH1 Array

Whole-genome tiling arrays allow for the comprehensive, genome-wide measurement of gene expression. However, because the Tiling 1.0F array has been developed only recently, few studies that use it to measure gene expression have been published. In contrast, the Genechip *Arabidopsis* ATH1 Genome array (Affymetrix), containing 22,500 probe sets that match to 23,688 genes, is a widely used tool to measure gene expression in *Arabidopsis*. Two recent studies used the ATH1 microarray to identify genes that are downregulated in ovules that lack embryo sacs: Yu et al. identified 249 genes (representing 225 probe sets) downregulated in *sporocyteless*/*nozzle* (*spl*/*nzz*) ovules [23], and Steffen et al*.* identified 104 genes (representing 86 probe sets) downregulated in *dif1* ovules [22]. A comparison to these datasets illustrates the utility of using genome-wide expression measures to profile gene expression and also validates the sensitivity of the whole genome tiling array as a means of quantifying gene expression.

Only 31% of the *dif1* downregulated genes identified by the whole genome tiling array analysis were reported as embryo sac–dependent by one or both studies using the ATH1 array (Figure S4). The large number of *DIF1*-dependent genes uniquely discovered by the tiling array is primarily due to the fact that a surprisingly large number of embryo sac–dependent genes were not measured by the ATH1 array. While 84% of all currently annotated *Arabidopsis* protein-coding genes had a corresponding probe set on the ATH1 array (at least six of 11 probes perfectly matching), a significantly smaller percentage (41%) of *DIF1*-dependent genes were represented by ATH1 probe sets (Table 4). In total, 224 *dif1* downregulated genes did not have ATH1 probe sets (Table 4).

**Table 4:**
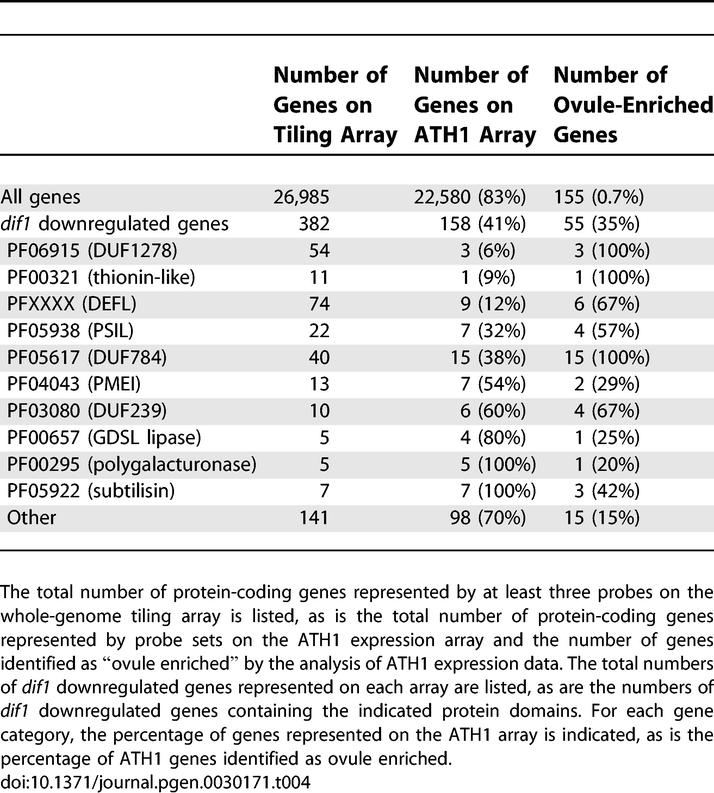
Gene Families Are Unevenly Represented on the ATH1 Array and among Ovule-Enriched Genes

In addition to failing to detect the majority of embryo sac–dependent genes, the ATH1 array is specifically biased against certain gene families. Whereas over 90% of *DIF1*-dependent genes encoding lipases, subtilisins, or polygalacturonases were represented in the ATH1 array, less than 20% of the *DIF1*-dependent genes belonging to families encoding small, functionally uncharacterized proteins (DUF784, DUF1278, DEFL, PSIL, and thionin-like genes) had corresponding ATH1 probe sets (Table 4; Figure S4). The poor representation of these families can be attributed to the fact that many members of these families were annotated after the design of the ATH1 array [29–31]. For example, 65 *DIF1*-dependent DUF784, DUF1278, and thionin-like genes were unannotated prior to the TAIR7 annotation release of April, 2007 [30]. The combined analysis of tiling array data and high-throughput cDNA sequencing led to the finding that large families of poorly understood, potentially secreted proteins are embryo sac dependent, a finding that was not evident from the more limited and biased sets of embryo sac–dependent genes detected by the ATH1 array.

Considering only those genes with ATH1 probe sets, there was considerable overlap between the tiling array and ATH1 data; 76% of the 158 *dif1* downregulated genes with ATH1 probe sets were reported as downregulated in at least one of the ATH1 analyses (Figure S4). Sixty-five genes were reported as downregulated in all three studies, and another 55 genes were reported as downregulated in both our analysis and in one of the other studies (Figure S4). Thus, the reproducibility and accuracy of gene expression quantification by the whole genome tiling array was at least roughly comparable to that of the more commonly used expression array.

### Many *SPL/NZZ*-Dependent Genes are *DIF1*-Independent

114 *spl*/*nzz* downregulated genes were not identified as downregulated in either study using *dif1* ovules (Figure S4.) Some of these genes were downregulated in *dif1* ovules, but at levels below our statistical thresholds. However, 69 *SPL*/*NZZ*-dependent genes were expressed at similar levels in *dif1* and wild-type ovules (*p* > 0.25) and seven were actually upregulated in *dif1* ovules (*p* < 0.05). The fact that a large number of *SPL*/*NZZ*-dependent genes are not downregulated in *dif1* ovules is most likely due to the different stages of ovule development at which *SPL*/*NZZ* and *DIF1* act. *SPL*/*NZZ* is known to be required for the proper expression of several key genes during development of the somatic ovule, and *spl*/*nzz* ovules never initiate meiosis [32–35]. In contrast, the somatic development of *dif1* ovules appears to be entirely wild type, and *dif1* ovules initiate meiosis properly [20]. Therefore, it seems that approximately one-fourth of *SPL*/*NZZ*-dependent genes are not embryo sac–dependent but rather require *SPL*/*NZZ* for expression in the somatic ovule.

### Many Embryo Sac–Dependent Genes Are Expressed Primarily within the Ovule

Although many embryo sac genes are not detected, the ATH1 array is capable of allowing quantitative comparisons to published studies using the same platform. We measured gene expression in three biological replicates from functionally wild-type ovules (from the male sterile *ms-1* mutant) on the ATH1 array. Normalized probe set expression values were calculated via the RMA method [36] from probe level data from the ovule arrays, together with probe level data from 41 sets of Affymetrix gene chip experiments from various wild-type tissues and developmental stages that did not contain stage 12 or later ovules [37,38]. We analyzed the data to identify genes for which (1) ovule expression was at least two times higher than that of any other tissue and (2) ovule expression was at least three standard deviations above the mean expression level in non-ovule tissues, resulting in 155 ovule-enriched genes (Table S10). Of the 158 *DIF1*-dependent genes with ATH1 probe sets, 55 were identified as ovule enriched (Table 4). Certain gene families were highly represented amongst the set of ovule-enriched genes, including all *DIF1*-dependent DUF784, DUF1278, and thionin-like genes with ATH1 probe sets, as well as the majority of *DIF1*-dependent DEFL and PSIL genes with ATH1 probe sets (Table 4). In combination, the ATH1 array data and RT-PCR data (Figure 3) show that numerous members of the DUF784, DUF1278, DEFL, and PSIL gene families are expressed primarily within the ovule.

### Expression of DUF784 Genes Requires *MYB98*

In contrast to the total ablation of embryo sac tissue in *dif1* ovules, *myb98* embryo sacs are morphologically similar to wild type with exception of the subcellular structure of the synergid cells [11]. *myb98* embryo sacs are also impaired in mediating pollen tube guidance [11]. Genes with reduced expression levels in *myb98* ovules are therefore likely to represent genes active during the final stages of embryo sac development and during the initial steps of the fertilization process.

**Figure 4:**
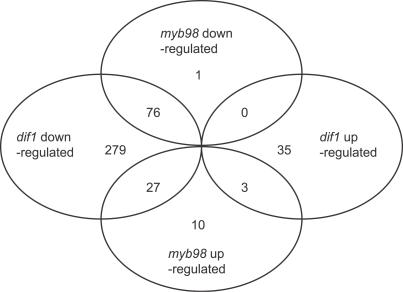
*myb98* Differentially Regulated Genes Are a Subset of *dif1* Differentially Regulated Genes The overlap of the sets of gene up and downregulated in *dif1* and *myb98* ovules is displayed in a venn diagram.

Using the same significance thresholds as in the *dif1* versus wild type comparison (*p* ≤ 0.001, log_2_ fold change > 1), we found that 77 genes were downregulated in *myb98* mutants compared to wild type, whereas 40 were upregulated (Tables 3, S7, and S8). As would be expected from the more severe *dif1* phenotype and the fact that *DIF1* is required for *MYB98* expression, the set of *MYB98*-dependent genes is largely a subset of the *DIF1*-dependent genes; 76 of the 77 *myb98* downregulated genes were also downregulated in *dif1* ovules (Figure 4). The set of *myb98* downregulated genes was even more highly enriched for genes encoding potentially secreted proteins (92%) and proteins weighing less than 20 kD (84%) than was the set of *dif1* downregulated genes (Table 3).

Thirty-seven of the 40 DUF784 genes encoded in the *Arabidopsis* genome were downregulated in *myb98* ovules (Table 3). In total, DUF784 genes comprised nearly 50% of the *myb98* downregulated genes, while other gene families overrepresented among *dif1* downregulated genes were represented to varying degrees among the *myb98* downregulated genes (Table 3). The magnitude of gene downregulation in *myb98* ovules was less than that in *dif1* ovules (Figure 2). Whereas all 40 DUF784 genes were downregulated at least 8-fold in *dif1* ovules as compared to wild type, only 23 were downregulated 8-fold in *myb98* ovules (Figure 2). No genes were downregulated 64-fold in *myb98* ovules.

RT-PCR analysis confirmed the degree of *myb98* dependence among the different gene families. Consistent with the microarray results, all of the 16 DUF784 genes tested were expressed at lower levels in *myb98* ovaries than in wild type, but many were detected at higher levels than in *dif1* ovaries (Figure 4). Also consistent with the array results, only a fraction of the DUF1278, PSIL, and DEFL genes tested were expressed at lower levels in *myb98* ovaries (Figure 3).

Of the 40 genes significantly upregulated in *myb98* ovules compared to wild type (Table 3), 27 were downregulated in *dif1* ovules (Figure 3). These appear to be genes that are expressed within the embryo sac, perhaps during the early stages of embryo sac development, and that go down in expression in response to *MYB98*. However, the fold increase amongst *myb98* upregulated genes was modest; only one was upregulated more than 4-fold relative to wild type (Figure 2).

### DUF784 and DUF1278 Genes Are Transcribed in Synergid Cells

**Figure 5:**
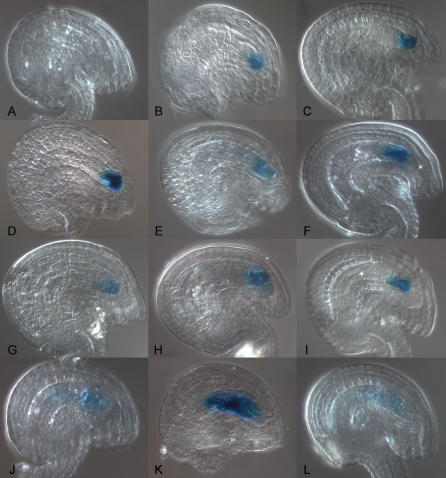
DUF784 and DUF1278 Genes Are Transcribed in Synergid Cells Stage 12c flowers were emasculated 24 h before ovules were stained for GUS expression. Ovules from plants transformed with promoterless GUS expression vector had no detectable GUS expression (A). Approximately 50% of ovules in T1 plants expressing GUS from one of four DUF784 promoters (B–E) or from one of four DUF1278 promoters (F–I) had a single locus of GUS expression near the micropyle, consistent with expression in the synergids, and in some cases possibly also the egg cell. Expression of GUS from a DEFL promoter (J) or one of two PSIL promoters (K, L) resulted in GUS expression within the central cell. (B) *At5g35405*::GUS, (C) *At4g08025*::GUS, (D) *At5g34885*::GUS, (E) *At2g21727*::GUS, (F) *At5g54062*::GUS, (G) *At2g14378*::GUS, (H) *At5g36340*::GUS, (I) *At5g42895*::GUS, (J) *At4g14276*::GUS, (K) *At4g24974*::GUS, and (L) *At5g27238*::GUS.

To localize the expression of *DIF1*-dependent and *MYB98*-dependent genes within the embryo sac, we constructed 11 transgenic lines expressing a glucouronisidase (GUS) reporter gene under the control of a putative promoter sequence corresponding to the genomic region upstream of an embryo sac–dependent gene. The four DUF784 promoters tested (*At5g35405*, *At4g08025*, *At5g34885*, and *At2g21727*) corresponded to genes that had large numbers of ovule cDNA reads (Table S4) and that represent different subfamilies of the DUF784 phylogenetic tree (Figure S2). In T1 plants of all four DUF784::GUS lines, ∼50% of ovules had a single, punctate spot of GUS expression located at the extreme micropylar end of the embryo sac (Figure 5A–5E). This localization of GUS expression is most consistent with transcription in the synergid cells, although in some cases GUS expression appeared to extend into the egg cell. GUS staining was observed in ovules of stage 12c flowers of DUF784::GUS plants, but not in ovules from stages 12b or earlier (unpublished data).

The four DUF1278 promoter::GUS lines analyzed also all resulted in GUS expression in synergid cells (Figure 5F–5I). Unlike the DUF784 promoters, all of which corresponded to *MYB98*-dependent genes, only two of the DUF1278 promoters tested (*At5g54062* and *At5g42895*) corresponded to *myb98* downregulated genes as determined by array analysis or RT-PCR. The synergid specific expression of *At5g36340*::GUS and *At2g24205*::GUS, neither of which correspond to *MYB98*-dependent genes, demonstrates that some synergid specific markers are *MYB98* independent.

In contrast to the expression in synergid cells observed for DUF784 and DUF1278 promoters, the DEFL promoter (Figure 5J) and PSIL promoters (Figure 5K and 5L) that were tested drove GUS expression within the central cell of the embryo sac.

### Embryo Sac–Dependent Gene Families Are Functionally Uncharacterized

Of the 382 *DIF1*-dependent genes, 241 (63%) belonged to one of ten gene families (Table 3). The subset of embryo sac–dependent genes that required the synergid-specific transcription factor *MYB98* was even more enriched (80%) for these same families (Table 3). While many of these gene families are similar in that they encode for small, potentially secreted proteins, each has a unique sequence profile, evolutionary history, and, presumably, role in embryo sac biology.

More than 20% of the embryo sac–dependent genes encoded for defensin-like or thionin-like proteins (Table 3), two classes of small, secreted proteins with disulfide-linked cysteines. Members of both classes are known to have antimicrobial or antifungal properties [27,28]. Not counting pseudogenes, there are approximately 286 defensin-like genes and 62 thionin-like genes in the *Arabidopsis* genome. For the vast majority of these genes, no functional or biochemical data exists [31]. We found that 32% of all *Arabidopsis* DEFL genes and 19% of all *Arabidopsis* thionin-like genes are embryo sac–dependent (Table 3). While the embryo sac–expressed genes of these families have homology to antipathogenic peptides, it is unknown whether the role of these families in the embryo sac is related to defense against pathogens or whether they serve other roles as small secreted proteins.

22 embryo sac–dependent genes, including seven annotated in this work, have homology to the S1 self-incompatibility protein of the genus *Papaver*. In *Papaver*, pistil-secreted S1 proteins inhibit growth and trigger cell death of incompatible pollen [39]. While Arabidopsis thaliana is self-fertile, other Brassicaceae, including the near relative Arabidopsis lyrata, exhibit self-incompatibility, albeit through incompatibility factors that do not resemble those in the *Papaver* stigma [26]. We found that 40% of PSIL genes encoded in the *Arabidopsis* genome are embryo sac–dependent. Data from the AtGenExpress expression atlas [37] show that most PSIL genes that are not embryo sac–dependent are expressed most highly in anthers or pollen, suggesting that PSIL genes also play a role in the male gametophyte.

The DUF784 and DUF1278 gene families are unique in that the majority of genes belonging to these two families are embryo sac–dependent (Table 3). In the case of DUF784, all 40 genes encoded in the genome are downregulated in *dif1* ovules, and 37 out of 40 are downregulated in *myb98* ovules (Table 3). Neither family has apparent homology to any protein with a known molecular or biological function, although the DUF1278 genes are related to the *EARLY CULTURE ABUNDANT1* gene identified in barley microspores [40] and to the EC1 gene that is expressed in wheat egg cells [41]. Both families are defined by the presence of six highly conserved cysteines that are present in almost all family members (Figures S2 and S3). While the pattern of conserved cysteines amongst DUF1278 proteins appears to be similar to that of DUF784, sequence homology between members of these two families was not detected by blastp, nor did HMMer searches using a HMM from one family find significant homology to members of the other family.

### DUF784 Genes Are Potential Mediators of Interactions between the Embryo Sac and Pollen Tubes

While several gene families were overrepresented among the set embryo sac–expressed genes, the extent of embryo sac specificity amongst DUF784 genes is particularly striking. All 40 genes belonging to this family were down regulated at least 8-fold in *dif1* ovules, and no DUF784 gene detected by the ATH1 array or tested by RT-PCR showed high levels of expression in any tissue outside of the ovule. Furthermore, all four of the DUF784 genes tested were transcribed within the synergid, and most DUF784 genes are significantly downregulated in *myb98* ovules. In total, DUF784 genes accounted for ∼50% of the *myb98* downregulated genes. The fact that the DUF784 family is both synergid expressed and *MYB98* dependent suggests that the pollen tube guidance defect in *myb98* ovules may be due to the downregulation of DUF784 genes and implicates the DUF784 family as being important for the development of pollen tube–attraction competence in synergid cells or as potential signaling molecules that are perceived directly by pollen tubes.

### Implications for Embryo Sac Development and Reproduction

Despite decades of research in plant sexual reproduction, the genetic mechanisms that underlie the development of the embryo sac and the interactions between the embryo sac and the pollen tube have remained poorly characterized. Through the use of truly genome-wide measures of gene expression that are capable of detecting unannotated genes and recently annotated genes, our analysis uncovered the embryo sac–dependent expression of hundreds of genes not analyzed by recent studies using annotation-based microarrays [22,23]. The finding that the majority of embryo sac–dependent genes are functionally uncharacterized underscores the limited extent of our understanding of embryo sac molecular biology. The finding that hundreds of embryo sac–dependent proteins are potentially secreted suggests that the number and complexity of intracellular communications, cell well modifications, and other extracellular events that take place during embryo sac development, fertilization, and the initiation of seed development may be even greater than previously realized.

We find that hundreds of related genes, comprising entire families and subfamilies of genes with unknown function, require the mature embryo sac for expression in ovules. The fact that so many paralagous genes have overlapping domains of expression in the embryo sac suggests that there is a high degree of functional redundancy between embryo sac genes. The coexpression of functionally redundant paralogs may explain why genes from these families have not been identified in forward genetic screens for female gametophytic mutants. Furthermore, many of these embryo sac–dependent genes are not expressed at high levels in tissues other than ovules, suggesting that they may be specialized for roles in female reproductive development and function. Future experiments to discover the potentially overlapping functions of embryo sac–dependent gene families will likely be crucial to building a more complete understanding of the genetic mechanisms that underlie plant sexual reproduction.

## Materials and Methods

### Plant growth and material.

Seeds for *ms-1* (CS75, Landsberg background), *dif1* (SALK_091193, Columbia background), and *myb98–1* (SALK_020263, Columbia background) were obtained from the Arabidopsis thaliana Biological Resources Center. Plants were grown in a growth chamber under long day (16 h light/8 h dark) conditions at 22 °C.

### High-throughput sequencing of ovule cDNAs.

Total RNA (0.5 μg)from stage 14 *ms-1* ovules was reverse transcribed and PCR amplified for 15 cycles using the BD SMART cDNA synthesis kit (Clontech) as per the manufacturer's instructions. The cDNA was fragmented and subjected to high-throughput 454 sequencing (454 Life Sciences) [24].

Primer sequence in the 454 reads was masked with Crossmatch (http://www.phrap.org), and each read was aligned to the *Arabidopsis* genome (January 2004 release) using blat with default settings [42]. Reads that had no blat hits were aligned to the genome with blastn (http://blast.wustl.edu) (parameters S = 100, S2 = 5, gapS2 = 200, X = 26, gapX = 55, W = 12, gapW = 18, gapall, Q = 11, R = 11, M = 5, N = −11, Z = 3e9, Y = 3e9, V = 1e6, B = 1e6, hspmax = 1000, hspsepqmax = 2e5, topcomboN = 200, wordmask = seg, maskextra = 10, hspsepsmax = 2000). For each match found by blat or blastn, more precise exon–exon boundaries were defined by running exalin [43] on the genomic region found by blat or blastn, with an additional 200 nucleotides of flanking sequence on each side. For each read, matches with submaximal exalin scores were discarded, as were matches which contained less than 20 aligning nucleotides, were composed of primarily (>75%) of a single nucleotide (usually A or T), or for which the read had less than 80% identity when compared to genomic sequence. Matches with overlapping genomic coordinates and which were not transcribed from opposite strands were grouped together to build consensus “contigs.” The genomic coordinates of matches to cDNA reads were compared to those of annotated genes (TAIR release 7). Each gene was assigned a normalized number of reads, where each match to a read was weighted relative to the number of genomic matches that read had (i.e., a match to read with a unique genomic match was given a weight of 1, whereas each match to a read with four equally good genomic matches was give a weight of 0.25).

### Whole-genome tiling array sample preparation and signal normalization.

Ovules were dissected from approximately one-month-old wild-type (Columbia ecotype), *dif1*, and *myb98* plants. To obtain mature, unpollinated ovules, stage 12a flowers were emasculated 24 h before collection of ovules. RNA was purified by RNAqueous-micro spin columns (Ambion). Sufficient material was collected to yield at least 2 μg of total RNA (requiring ∼1600 ovules from ∼40 ovaries) for each of four biological replicate for each genotype (i.e., 12 samples total). Preparation of samples for hybridization to tiling arrays was carried out as previously described [44]. PolyA RNA was purified with Oligotex beads (Qiagen), and random hexamer-primed first-strand cDNA was reverse transcribed with Superscript III reverse transcriptase (Invitrogen) at 42 °C for 1 h. Second-strand cDNA was synthesized in second-strand reaction buffer (Invitrogen) with 40 U of E. coli DNA polymerase I (New England Biolabs), 10 U of E. coli DNA ligase (New England Biolabs), and 2 U of E. coli RNase H (Epicentre) at 16 °C for 2 h. cDNA samples were incubated with 10 U of RNase H, 0.5 U of RNaseA, and 20 U of RNaseT1 at 37 °C for 20 min and then purified on Qiaquick spin columns (Qiagen). Samples were biotin labeled using the Bioprime system (Invitrogen) and concentrated by ethanol precipitation. Samples were hybridized to Genechip *Arabidopsis* Tiling 1.0F arrays (Affymetrix) and probe intensities were scanned at the University of Chicago Functional Genomics Center. Expression data was analyzed using the R Project for Statistical Computing and the affy package [45]. Probe intensities were corrected for spatial abnormalities [46], the perfect match intensities were background corrected with the bg.adjust function of the affy package, and the log2 transformed perfect match intensities were quantile normalized across the 12 arrays.

### De novo identification of *DIF1*-dependent intervals.

All 25mer probes on the *Arabidopsis* Tiling 1.0F array were matched to the *Arabidopsis* genome using blastn. Probes perfectly matching the genome more than 30 times were removed from the analysis. For each probe match, a *p-*value and mean log_2_ fold change were calculated by a *t-*test comparing the expression values of the four replicate wild-type expression values against the expression values of the four *dif1* replicates. Probes with log_2_ fold changes less than 1 or *p-*values greater than 0.05 were removed from the analysis. Probe matches that passed these thresholds and that were located within 85 bp of each other were iteratively grouped together to define differentially expressed intervals. Intervals containing less than three passing probe matches were removed from the analysis.

### Discovery of embryo sac–dependent unannotated protein coding genes.

The set of *dif1* downregulated genomic intervals was compared to genomic locations of existing gene annotations (TAIR release 7) to identify those not mapping to within 50 bp of an annotated gene. Intergenic intervals that overlapped the genomic alignments of ovule 454 cDNA contigs were considered as potential gene fragments. Adjacent contigs were considered to be part of the same gene if they were within 200 bp or overlapped the same cDNA contig. ORFs were predicted by extending the longest ORF within the interval into flanking genomic sequence. In cases where cDNA reads suggested splicing, the splice sites of the cDNA reads were used to guide ORF annotation.

### Analysis of differential expression.

The positions of probe matches were compared to the positions of annotated exons (TAIR7 release) and to the positions of newly identified protein coding genes. Probe matches that overlapped with more than one gene (i.e., regions for which both strands are transcribed) were removed from the analysis, as were genes having less than three probe matches. For each gene, the mean log_2_ fold change and corresponding *p-*value was calculated from a *t-*test of the wild-type expression values against the mutant expression values (*dif1* or *myb98*) across all probes matching that gene. Genes were considered to be differentially regulated if the *p-*value was less than 0.001 and the log_2_ change in expression was greater than 1. The FDR at this threshold was estimated by permuting the groupings of the four wild-type and four *dif1* arrays. The set of eight arrays was partitioned into two “balanced” groups of four such that each group of four contained two wild-type arrays and two *dif1* arrays. The expression data was reanalyzed for each of 18 possible “balanced” permutations of array groupings, and the FDR estimated as the average number of genes passing the statistical thresholds for the permuted groupings as a percentage of the number passing the thresholds for the actual grouping of arrays. The decision to base FDR estimate on the 18 balanced permutations, rather than on all 35 possible permutation (including the actual permutation), was based on the observation that the large number of genes highly downregulated in *dif1* ovules resulted in an unreasonably strict threshold when the nonbalanced permutations were included in the FDR analysis.

### Analysis of protein characteristics.

The list of PFAM domains in TAIR7 proteins was downloaded the TAIR website. For the DEFL, DUF784, DUF1278, and thionin-like families, the existing PFAM annotations were found to omit family members. The set of nonpseudogene DEFL genes was taken from Silverstein et al. [31]. For the DUF784 and DUF1278 families, the HMMs downloaded from the PFAM website were used to iteratively search the annotated peptides using HMMer (version 3.2, http://hmmer.janelia.org/) to identify additional family members. Most genes annotated as “plant thionins” were found not to correspond to the “plant thionin” (PF00321) PFAM domain. An HMM was built using all proteins annotated as encoding a “thionin” or “thionin-like” protein, which was then used to search for additional family members. The genes found by this HMMer search are referred to as “thionin-like” in this paper.

Gene families were considered to be overrepresented in the set of *dif1* downregulated genes if at least five *dif1* downregulated genes encoded that domain and the number of down regulated gene encoding that domain was significantly greater than the expected number (based on the frequency of that domain amongst all proteins) as determined by a chi-squared test. The presence of putative signal peptides was predicted with SignalP [47].

### Comparison of embryo sac–dependent gene sets.

To allow for the comparison of previously published gene sets from studies using the ATH1 array, we mapped ATH1 probe sets to the most recent gene annotations (TAIR7) by blasting the probe sequences against the annotated cDNA sequences. A probe set was considered to match a gene if at least six of the 11 probes perfectly matched that gene. Previously published sets of *SPL/NZZ*-dependent genes [23] and *DIF1*-dependent genes [22] were retabulated based on the mappings of the published ATH1 probe sets to the TAIR7 annotations. In cases for which a single ATH1 probe set mapped to multiple genes, all matched genes were considered as downregulated for purposes of comparison to the whole-genome tiling array data.

### ATH1 Genechip analysis.

Ovules were microdissected out of mature (∼stage 14) ovaries from *ms1*/*ms1* homozygotes (Landsberg ecotype), and RNA was purified using RNeasy columns (Qiagen) as per the manufacturer's instructions. Three replicate samples were collected, each of which yielded sufficient RNA (>7 μg) to allow unamplified preparation of cRNA for Affymetrix analysis. Preparation of labeled cRNA, hybridizations to ATH1 Genechip arrays (Affymetrix), and scanning of probe-level scores were carried out at the Keck Foundation Biotechnology Resource Laboratory (Yale University, New Haven, Connecticut). Probe level data from the three ovule arrays, along with probe level data from multiple tissues and developmental stages contained in AtGenExpress (samples ATGE_1, ATGE_10, ATGE_12, ATGE_13, ATGE_14, ATGE_15, ATGE_16, ATGE_17, ATGE_2, ATGE_24, ATGE_25, ATGE_26, ATGE_27, ATGE_28, ATGE_29, ATGE_3, ATGE_31, ATGE_32, ATGE_34, ATGE_35, ATGE_36, ATGE_4, ATGE_40, ATGE_41, ATGE_42, ATGE_43, ATGE_5, ATGE_6, ATGE_73, ATGE_76, ATGE_77, ATGE_78, ATGE_79, ATGE_8, ATGE_81, ATGE_82, ATGE_83, ATGE_84, and ATGE_9) [37], as well as seedling and stigma data from Swanson et al. [38], were normalized using the RMA method [36] as implemented in the affy R package [45], and the mean expression value was calculated for each probe set in each tissue type. The “ovule-specificity factor” (Ov_sp_) was calculated for each probe set as Ov_sp_ = (Exp_ovule_ − Exp_mean_)/SD, where Exp_ovule_ is the mean expression in stage 14 ovules, Exp_mean_ is the mean log_2_ expression value across all tissue types (excluding those that contain stage 12 to stage 14 ovules), and SD is the standard deviation of mean expression values across all tissue types (again excluding tissues containing stage12 or stage 14 ovules). ATH1 probe sets were mapped to the most recent gene annotations (TAIR7) by blasting the probe sequences against the annotated cDNA sequences. A probe set was considered to match a gene if at least six of the 11 probes perfectly matched that gene.

### RT-PCR.

Samples from different tissues and developmental stages were collected from 1-month-old *ms-1* plants, except for anther samples, which were collected from wild-type Landsberg plants. For wild-type Columbia, *dif1*, and *myb98* ovary samples, stage 12a flowers were emasculated 24 h before ovary tissue was collected. RNA was purified by RNeasy spin columns (Qiagen). For each tissue, 0.5 μg of RNA was treated with DNase before first-strand cDNA was reverse transcribed using Protoscript II RT-PCR kit (New England Biolabs) and diluted to 100 μl. Each RT-PCR reaction used 2 μl of first-strand cDNA (or 2 μl of no reverse transcriptase control) as template in a 30 μl reaction with 0.33 μM of each primer. Primer sequences are contained in Table S11.

### Analysis of promoter::GUS fusions.

Genomic sequences upstream of embryo sac–dependent genes were PCR amplified and cloned into pCAMBIA-1381Z upstream of the GUS ORF (Table S12). Arabidopsis thaliana plants (Columbia ecotype) were transformed by the floral dip method [48]. Seeds were grown on MS media containing 25 mg/ml hygromycin for 12 d before seedlings with true leaves were transferred to soil. Stage 12c flowers were emasculated 24 h before ovules were dissected into GUS staining solution (50 mM sodium phosphate buffer pH 7.0, 10 mM EDTA, 0.1% Triton X-100, 2 mM potassium ferrocyanide, 2 mM potassium ferricyanide, and 1 mg/ml X-Gluc) on ice before incubation at 37 °C for 45 min (12 h for *At5g34885*::GUS and *At4g24974*::GUS*)*. Samples were cleared in 20% methanol/4% concentrated HCl at 55 °C for 15 min followed by 60% ethanol/1.8 M NaOH at 25 °C for 10 min. Samples were washed with 30% ethanol and 10% ethanol and transferred to 50% glycerol for mounting on slides. Samples were viewed on a Zeiss Axioscope using DIC optics, and images were captured on a Zeiss AxioCam HRc digital camera.

## Supporting Information

Dataset S1 Genomic Coordinates of Alignments of Ovule cDNA Reads (13.4 MB TDS)

Figure S1 Distribution of Ovule cDNA Reads Per Gene The distribution of the number of ovule cDNAs mapping to each TAIR7 gene is plotted as a histogram. Genes with no reads are not included.
(477 KB PDF)

Figure S2 Alignment and Phylogenetic Tree of DUF784 Gene Family (A) Alignment of the 40 DUF784 proteins encoded in the *Arabidopsis* genome. Shaded residues are conserved in at least 90% of family members. The portion of the alignment containing highly conserved amino acids is shown.
(B) Neighbor-joining phylogenetic tree of the 40 *Arabidopsis* DUF784 proteins. Boot strap values ≥90% are shown.
(790 KB PDF)

Figure S3 Alignment and Phylogenetic Tree of DUF1278 Gene Family Alignment of 54 DUF1278 genes that are downregulated in *dif1* ovules. Shaded residues are conserved in at least 85% of aligned proteins. The portion of the alignment containing highly conserved amino acids is shown.
(B) Neighbor-joining phylogenetic tree of the 54 *dif1* downregulated aligned DUF1278 genes. Boot strap values ≥90% are shown.
(794 KB PDF)

Figure S4 Comparison to Embryo Sac–Dependent Genes Identified in Other Studies The overlap of the sets of genes reported as downregulated in mutant ovules in this study (*dif1* ovules [Columbia background], whole-genome tiling array), Steffen et al. [22] (*dif1* ovules [Landsberg background], ATH1 array), and Yu et al. [23] (*spl*/*nzz* ovules, ATH1 array) are displayed as a Venn diagram. For each subset, the total number of protein-coding genes is indicated, followed, in parentheses, by the number of genes encoding proteins predicted to weigh less than 20 kD and contain signal peptides. The gene sets from Steffen et al. and Yu et al. were defined based on our mapping of the published ATH1 probes sets to TAIR7 gene annotations (see Materials and Methods).
(462 KB PDF)

Table S1 Correlation Coefficients between Whole-Genome Tiling Arrays The correlation coefficients between quantile normalized whole-genome tiling arrays are shown.
(75 KB DOC)

Table S2 Estimated FDRs for *DIF1*-Dependent Genes at Different Statistical Thresholds The numbers of genes (including pseudogenes) that surpass the indicated *p-*value and log_2_ change cutoffs for the actual values of the wild-type and *dif1* arrays are shown, as are the average numbers of genes passing each set of cutoffs across the 18 balanced permutations of wild-type and *dif1* datasets. The FDR is estimated as the as average “differentially expressed” genes for the permuted datasets as a percentage of the number of genes differentially expressed for the actual dataset.
(68 KB DOC)

Table S3 Sequences and Genomic Coordinates of Newly Annotated *DIF1*-Dependent Genes (22 KB XLS)

Table S4 Expression Levels of All Nonpseudogenes (11.4 MB XLS)

Table S5 *dif1* Downregulated Genes (169 KB XLS)

Table S6 *dif1* Upregulated Genes (29 KB XLS)

Table S7 *myb98* Downregulated Genes (44 KB XLS)

Table S8 *myb98* Upregulated genes (29 KB XLS)

Table S9 *dif1* Downregulated pseudogenes (24 KB XLS)

Table S10 Ovule-Enriched Genes (67 KB XLS)

Table S11 RT-PCR Primer Sequences (18 KB XLS)

Table S12 Sequences of GUS Promoter Fusion Constructs (25 KB XLS)

### Accession Numbers

Raw and processed microarray data is available at the National Center for Biotechnology Information (NCBI) Gene Expression Omnibus (http://www.ncbi.nlm.nih.gov/geo/), with the series identifier GSE8392. Sequences of ovule cDNA reads are available at NCBI dbEST (http://www.ncbi.nlm.nih.gov/dbEST/), with the identifier numbers 45453167–45702604.

## Abbreviations

DEFL: defensin-like
DUF: domain of unknown function
FDR: false discovery rate
GUS: glucouronisidase
ORF: open reading frame
PMEI: pectinmethylesterase inhibitor
PSIL: Papaver self-incompatibility protein-like
RT-PCR: reverse transcriptase PCR
TAIR: The Arabidopsis Information Resource
