# Supplementary material for: Genome-Wide Expression Profiling of the Arabidopsis Female Gametophyte Identifies Families of Small, Secreted Proteins
Source: PLoS Genet. 2007 Oct 12;3(10):e171. doi: 10.1371/journal.pgen.0030171 (PMC2014789; doi:10.1371/journal.pgen.0030171)
Supplement: Supplementary file 1 [file pgen.0030171.sg001.pdf]

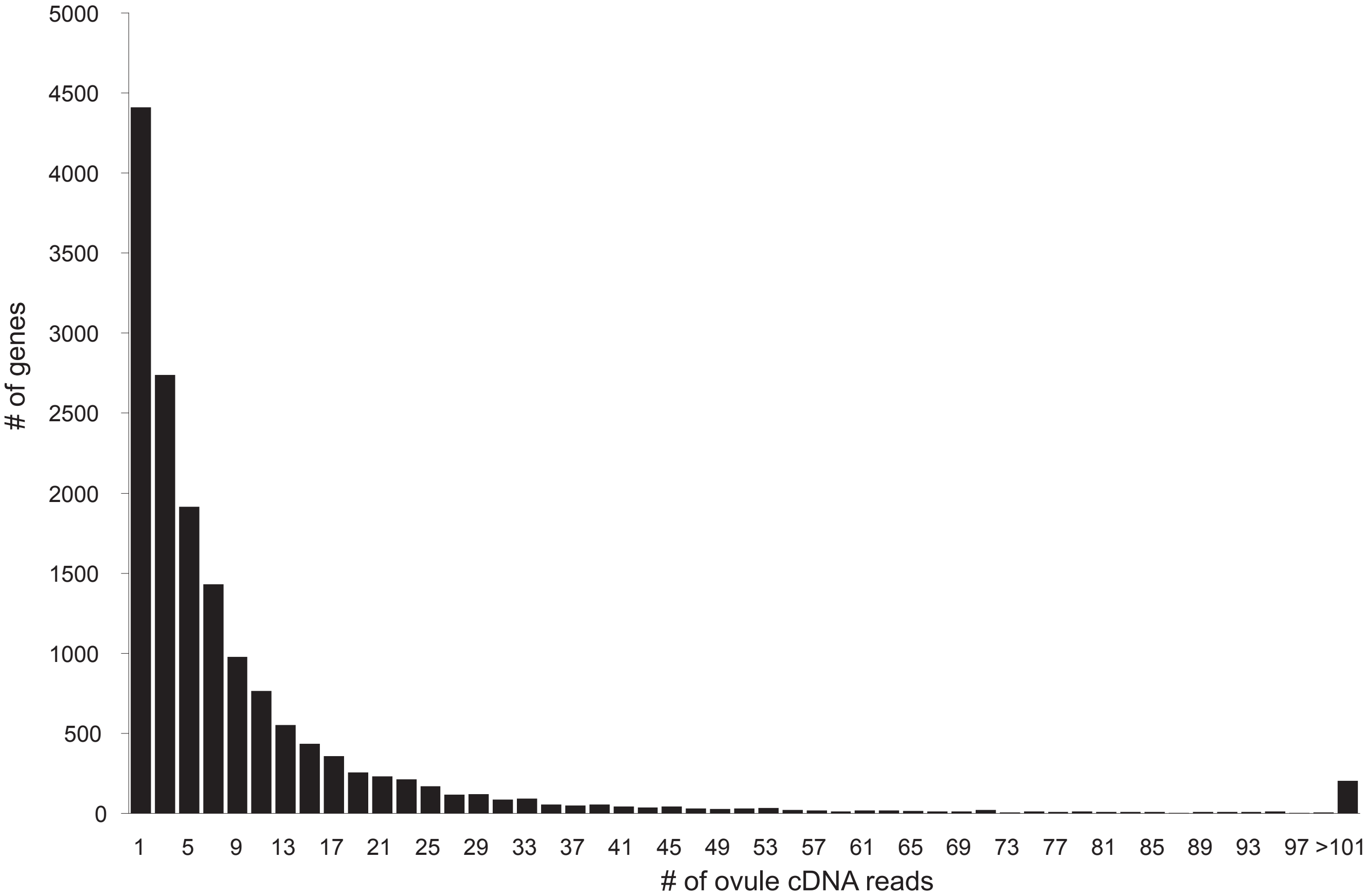

5000
4500
4000
3500
3000
2500
2000
1500
1000
500
0
# of genes
1
5
9
13
17
21
25
29
33
37
41
45
49
53
57
61
65
69
73
77
81
85
89
93
97
>101
# of ovule cDNA reads
