# Supplementary material for: Genome-Wide Expression Profiling of the Arabidopsis Female Gametophyte Identifies Families of Small, Secreted Proteins
Source: PLoS Genet. 2007 Oct 12;3(10):e171. doi: 10.1371/journal.pgen.0030171 (PMC2014789; doi:10.1371/journal.pgen.0030171)
Supplement: Supplementary file 2 [file pgen.0030171.sg002.pdf]

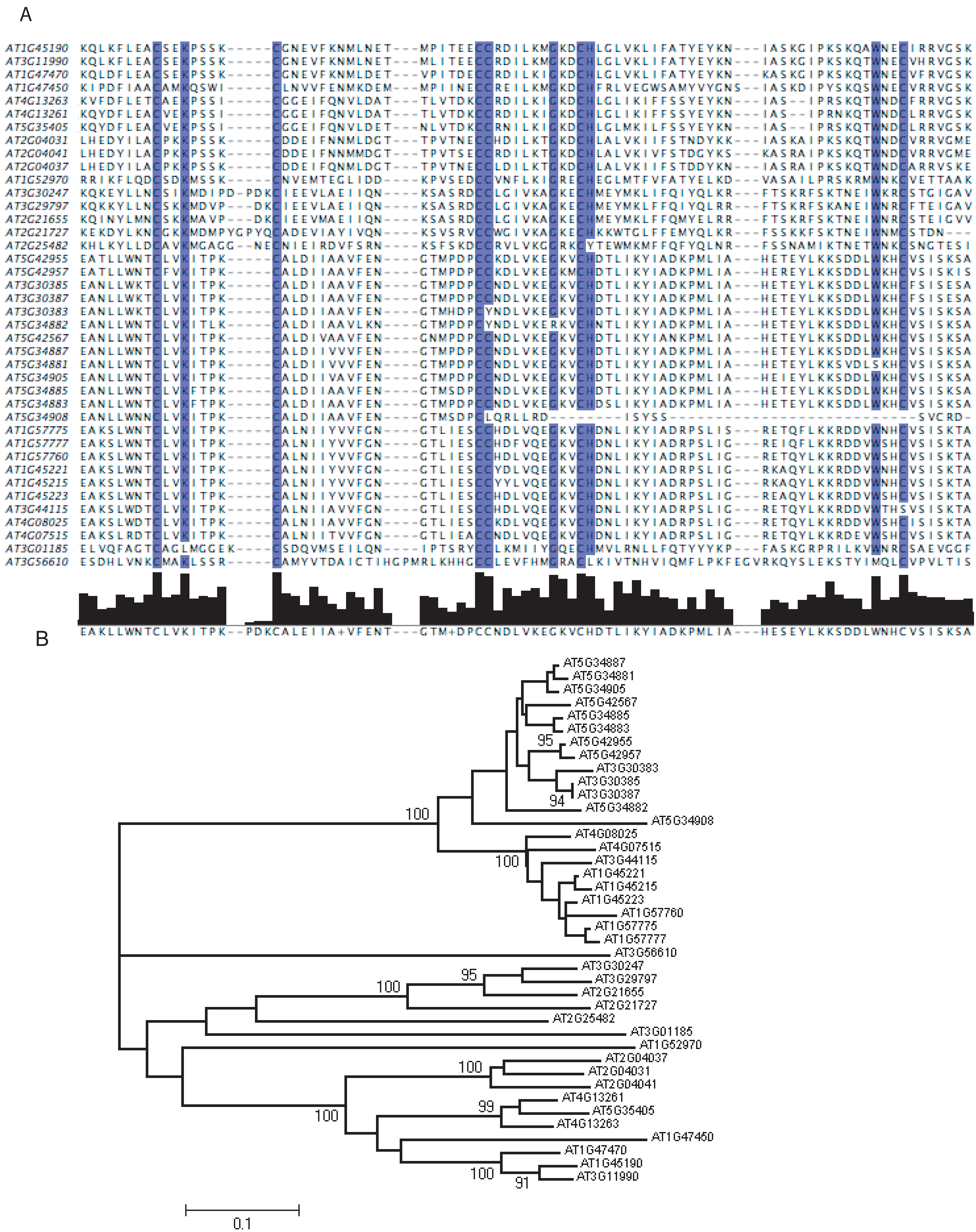
A
AT1G45190
AT3G11990
AT1G47470
AT1G47450
AT4G13263
AT4G13261
AT5G35405
AT2G04031
AT2G04041
AT2G04037
AT1G52970
AT3G30247
AT3G29797
AT2G21655
AT2G21727
AT2G25482
AT5G42955
AT5G42957
AT3G30385
AT3G30387
AT3G30383
AT5G34882
AT5G42567
AT5G34887
AT5G34881
AT5G34905
AT5G34885
AT5G34883
AT5G34908
AT1G57775
AT1G57777
AT1G57760
AT1G45221
AT1G45215
AT1G45223
AT3G44115
AT4G08025
AT4G07515
AT3G01185
AT3G56610
B
AT5G34887
AT5G34881
AT5G34905
AT5G42567
AT5G34885
AT5G34883
95
AT5G42955
AT5G42957
AT3G30383
AT3G30385
AT3G30387
94
AT5G34882
100
AT5G34908
AT4G08025
AT4G07515
100
AT3G44115
AT1G45221
AT1G45215
AT1G45223
AT1G57760
AT1G57775
AT1G57777
AT3G56610
AT3G30247
95
AT3G29797
100
AT2G21655
AT2G21727
AT2G25482
AT3G01185
AT1G52970
AT2G04037
100
AT2G04031
AT2G04041
AT4G13261
99
AT5G35405
100
AT4G13263
AT1G47450
AT1G47470
AT1G45190
100
91
AT3G11990
0.1
