# Supplementary material for: Genome-Wide Expression Profiling of the Arabidopsis Female Gametophyte Identifies Families of Small, Secreted Proteins
Source: PLoS Genet. 2007 Oct 12;3(10):e171. doi: 10.1371/journal.pgen.0030171 (PMC2014789; doi:10.1371/journal.pgen.0030171)
Supplement: Supplementary file 3 [file pgen.0030171.sg003.pdf]

A

B

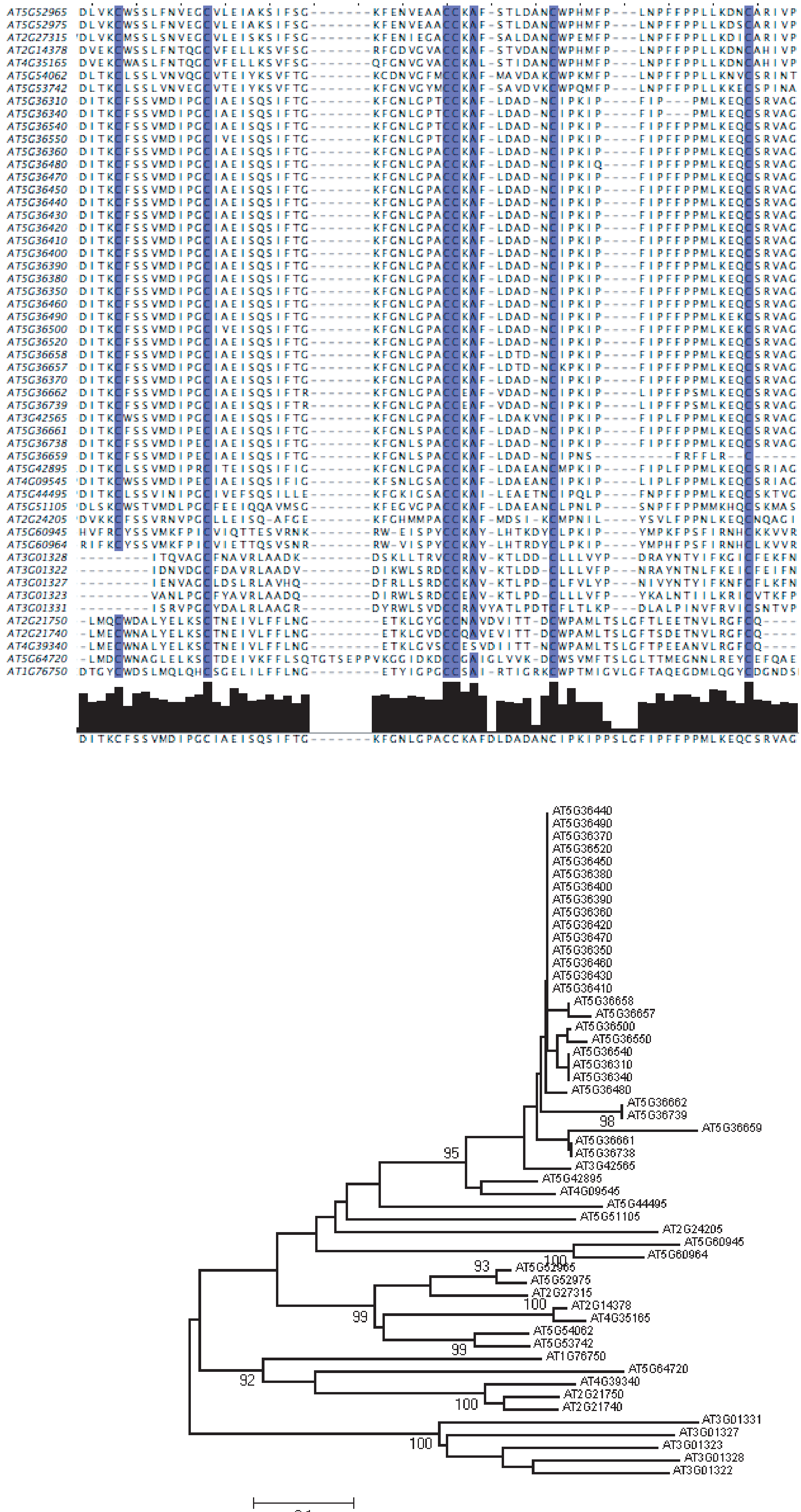
AT5G52965
AT5G52975
AT2G27315
AT2G14378
AT4G35165
AT5G54062
AT5G53742
AT5G36310
AT5G36340
AT5G36540
AT5G36550
AT5G36360
AT5G36480
AT5G36470
AT5G36450
AT5G36440
AT5G36430
AT5G36420
AT5G36410
AT5G36400
AT5G36390
AT5G36380
AT5G36350
AT5G36460
AT5G36490
AT5G36500
AT5G36520
AT5G36658
AT5G36657
AT5G36370
AT5G36662
AT5G36739
AT3G42565
AT5G36661
AT5G36738
AT5G36659
AT5G42895
AT4G09545
AT5G44495
AT5G51105
AT2G24205
AT5G60945
AT5G60964
AT3G01328
AT3G01322
AT3G01327
AT3G01323
AT3G01331
AT2G21750
AT2G21740
AT4G39340
AT5G64720
AT1G76750
98
95
100
93
99
100
99
92
100
100
0.1
