# Supplementary material for: Genome-Wide Expression Profiling of the Arabidopsis Female Gametophyte Identifies Families of Small, Secreted Proteins
Source: PLoS Genet. 2007 Oct 12;3(10):e171. doi: 10.1371/journal.pgen.0030171 (PMC2014789; doi:10.1371/journal.pgen.0030171)
Supplement: Supplementary file 4 [file pgen.0030171.sg004.pdf]

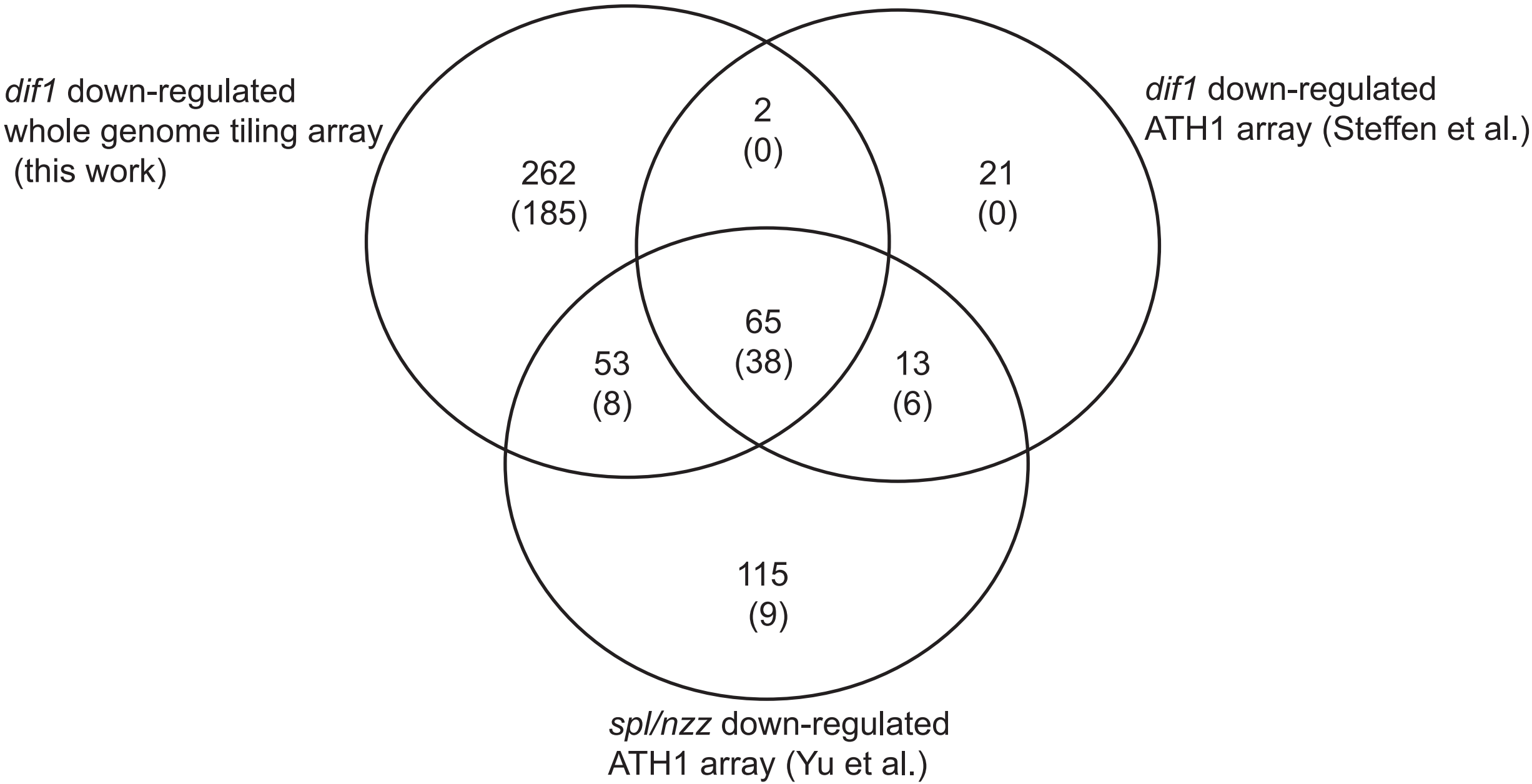
dif1 down-regulated
whole genome tiling array
(this work)
dif1 down-regulated
ATH1 array (Steffen et al.)
262
(185)
2
(0)
21
(0)
53
(8)
65
(38)
13
(6)
115
(9)
spl/nzz down-regulated
ATH1 array (Yu et al.)
