# Supplementary material for: Genome-Wide Expression Profiling of the Arabidopsis Female Gametophyte Identifies Families of Small, Secreted Proteins
Source: PLoS Genet. 2007 Oct 12;3(10):e171. doi: 10.1371/journal.pgen.0030171 (PMC2014789; doi:10.1371/journal.pgen.0030171)
Supplement: Supplementary file 5 [file pgen.0030171.st001.doc]

| Table S1. Correlation coefficients between whole genome tiling arrays | | | | | | | | | | | | |
| --- | --- | --- | --- | --- | --- | --- | --- | --- | --- | --- | --- | --- |
|  | Col-1 | Col-2 | Col-3 | Col-4 | dif-1 | dif-2 | dif-3 | dif-4 | myb-1 | myb-2 | myb-3 | myb-4 |
| Col-1 | 1.000 | 0.970 | 0.970 | 0.971 | 0.953 | 0.966 | 0.963 | 0.966 | 0.971 | 0.971 | 0.969 | 0.966 |
| Col-2 |  | 1.000 | 0.972 | 0.971 | 0.951 | 0.967 | 0.967 | 0.970 | 0.973 | 0.975 | 0.964 | 0.974 |
| Col-3 |  |  | 1.000 | 0.970 | 0.952 | 0.966 | 0.961 | 0.968 | 0.971 | 0.974 | 0.969 | 0.972 |
| Col-4 |  |  |  | 1.000 | 0.954 | 0.966 | 0.963 | 0.967 | 0.972 | 0.972 | 0.968 | 0.968 |
| dif-1 |  |  |  |  | 1.000 | 0.958 | 0.953 | 0.957 | 0.957 | 0.955 | 0.957 | 0.952 |
| dif-2 |  |  |  |  |  | 1.000 | 0.965 | 0.968 | 0.968 | 0.968 | 0.965 | 0.965 |
| dif-3 |  |  |  |  |  |  | 1.000 | 0.967 | 0.967 | 0.967 | 0.958 | 0.964 |
| dif-4 |  |  |  |  |  |  |  | 1.000 | 0.969 | 0.970 | 0.963 | 0.968 |
| myb-1 |  |  |  |  |  |  |  |  | 1.000 | 0.977 | 0.972 | 0.973 |
| myb-2 |  |  |  |  |  |  |  |  |  | 1.000 | 0.971 | 0.976 |
| myb-3 |  |  |  |  |  |  |  |  |  |  | 1.000 | 0.967 |
| myb-4 |  |  |  |  |  |  |  |  |  |  |  | 1.000 |
| The correlation coefficients between quantile normalized whole genome tiling arrays are shown. | | | | | | | | | | | | |
