# Supplementary material for: Genome-Wide Expression Profiling of the Arabidopsis Female Gametophyte Identifies Families of Small, Secreted Proteins
Source: PLoS Genet. 2007 Oct 12;3(10):e171. doi: 10.1371/journal.pgen.0030171 (PMC2014789; doi:10.1371/journal.pgen.0030171)
Supplement: Supplementary file 6 [file pgen.0030171.st002.doc]

| Table S2. Estimated false discovery rates for *DIF1*-dependent genes at different statistical thresholds | | | | |
| --- | --- | --- | --- | --- |
| P | log_2_ change | differentially-expressed genes  actual permuted | | FDR |
| 0.05 | 0 | 1167 | 82.8 | 7.1% |
| 0.05 | 1 | 494 | 5.4 | 1.1% |
| 0.05 | 2 | 299 | 0.0 | 0.0% |
| 0.01 | 0 | 782 | 33.1 | 4.2% |
| 0.01 | 1 | 481 | 5.4 | 1.1% |
| 0.01 | 2 | 298 | 0.0 | 0.0% |
| 0.005 | 0 | 705 | 24.7 | 3.5% |
| 0.005 | 1 | 469 | 4.8 | 1.0% |
| 0.005 | 2 | 297 | 0.0 | 0.0% |
| 0.001 | 0 | 582 | 12.8 | 2.2% |
| 0.001 | 1 | 446 | 3.6 | 0.8% |
| 0.001 | 2 | 295 | 0.0 | 0.0% |
| 0.0005 | 0 | 545 | 10.6 | 1.9% |
| 0.0005 | 1 | 434 | 3.1 | 0.7% |
| 0.0005 | 2 | 294 | 0.0 | 0.0% |
| 0.0001 | 0 | 480 | 6.6 | 1.4% |
| 0.0001 | 1 | 412 | 2.3 | 0.6% |
| 0.0001 | 2 | 289 | 0.0 | 0.0% |
| The numbers of genes (including pseudogenes) that surpass the indicated P value and log_2_ change cutoffs for the actual values of the wild type and *dif1* arrays are shown, as are the average numbers of genes passing each set of cutoffs across the 18 balanced permutations of wild type and *dif1* datasets. The false discovery rate (FDR) is estimated as the as average “differentially expressed” genes for the permuted datasets as a percentage of the number of genes differentially expressed for the actual data set. | | | | |
